# Supplementary material for: Increased Risk of Hospitalization for Pneumonia in Italian Adults from 2010 to 2019: Scientific Evidence for a Call to Action
Source: Vaccines (Basel). 2023 Jan 16;11(1):187. doi: 10.3390/vaccines11010187 (PMC9862073; doi:10.3390/vaccines11010187)
Supplement: Supplementary file 1 [file vaccines-11-00187-s001.zip › vaccines-2111766-supplementary.pdf]

Table S1. ICD 9 CM codes used to identify hospitalizations for pneumonia.

- 480.0 Adenovirus pneumonia
- 480.1 Respiratory syncytial virus pneumonia
- 480.2 Parainfluenza virus pneumonia
- 480.3 SARS-associated Coronavirus pneumonia
- 480.8 Pneumonia due to other viruses, not elsewhere classified
- 480.9 Viral pneumonia, unspecified
- 481 Pneumococcal pneumonia (Streptococcal pneumoniae pneumonia)
- 482.0 Klebsiella pneumoniae pneumonia
- 482.1 Pseudomonas pneumonia
- 482.2 Hemophilus influenzae pneumonia (H. influenzae)
- 482.30 Streptococcal pneumonia, unspecified
- 482.31 Streptococcal pneumonia, group A
- 482.32 Streptococcal pneumonia, group B
- 482.39 Pneumonia due to other Streptococci
- 482.40 Staphylococcal pneumonia, unspecified
- 482.41 Staphylococcal aureus pneumonia
- 482.81 Pneumonia due to anaerobes
- 482.82 Escherichia coli pneumonia [E. coli]
- 482.83 Pneumonia due to other gram-negative bacteria
- 482.89 Pneumonia due to other specified bacteria
- 482.9 Bacterial pneumonia, unspecified
- 483.0 Mycoplasma Pneumoniae pneumonia
- 483.1 Chlamydial pneumonia
- 483.8 Pneumonia due to other specified organisms
- 484.1 Cytomegalovirus pneumonia
- 484.3 Pneumonia in whooping cough
- 484.6 Pneumonia in aspergillosis
- 484.7 Pneumonia in other systemic mycoses
- 484.8 Pneumonia in other infectious diseases classified elsewhere
- 485 Bronchopneumonia, unspecified agent
- 486 Pneumonia, unspecified agent
- 487.0 Influenza with pneumonia

Table S2. ICD 9 CM codes used to identify the different possible underlying comorbidities observed in each hospitalization

ICD 9 CM codes	Diagnosis	Etiologic class
480.0	Adenovirus pneumonia	Specified Pneumonia viral
480.1	Respiratory syncytial virus	Specified Pneumonia viral
480.2	Parainfluenza virus pneumonia	Specified Pneumonia viral
480.3	SARS-associated Coronavirus	Specified Pneumonia viral
480.8	Pneumonia due to other viruses,	Unspecified Pneumonia
480.9	Viral pneumonia, unspecified	Unspecified Pneumonia
481	Pneumococcal pneumonia	Specified Pneumonia
482.0	Klebsiella pneumoniae	Specified Pneumonia
482.1	Pseudomonas pneumonia	Specified Pneumonia
482.2	Hemophilus influenzae	Specified Pneumonia
482.30	Streptococcal pneumonia,	Specified Pneumonia
482.31	Streptococcal pneumonia, group	Specified Pneumonia
482.32	Streptococcal pneumonia, group	Specified Pneumonia
482.39	Pneumonia due to other	Specified Pneumonia
482.40	Staphylococcal pneumonia,	Specified Pneumonia
482.41	Staphylococcal aureus	Specified Pneumonia
482.8	Pneumonia due to anaerobes	Specified Pneumonia
482.82	Escherichia coli pneumonia [E.	Specified Pneumonia
482.83	Pneumonia due to other gram-	Unspecified Pneumonia
482.89	Pneumonia due to other	Specified Pneumonia
482.9	Bacterial pneumonia,	Unspecified Pneumonia
483.0	Mycoplasma Pneumoniae	Specified Pneumonia
483.1	Chlamydial pneumonia	Specified Pneumonia
483.8	Pneumonia due to other	Others
484.1	Cytomegalovirus pneumonia	Specified Pneumonia viral
484.3	Pneumonia in whooping cough	Specified Pneumonia
484.6	Pneumonia in aspergillosis	Specified Pneumonia fungi
484.7	Pneumonia in other systemic	Unspecified Pneumonia
484.8	Pneumonia in other infectious	Unspecified Pneumonia
485	Bronchopneumonia, unspecified	Unspecified Pneumonia
486	Pneumonia, unspecified agent	Unspecified Pneumonia
487.0	Influenza with pneumonia	Specified Pneumonia viral

Table S3. ICD 9 CM codes used to identify the different possible causes of pneumonia.

ICD9-CM	Description	Comorbidities
416.9	Chronic cardiopulmonary disease, unspecified	Chronic respiratory diseases
491.0	Simple chronic bronchitis	Chronic respiratory diseases
491.1	Chronic mucopurulent bronchitis	Chronic respiratory diseases
491.20	Chronic obstructive bronchitis, without exacerbation	Chronic respiratory diseases
491.21	Chronic obstructive bronchitis, with exacerbation (acute)	Chronic respiratory diseases
491.22	Chronic obstructive bronchitis with acute bronchitis	Chronic respiratory diseases
491.8	Other chronic bronchitis	Chronic respiratory diseases
491.9	Unspecified chronic bronchitis	Chronic respiratory diseases
493.20	Chronic obstructive asthma	Chronic respiratory diseases
493.22	Chronic obstructive asthma with flare-up	Chronic respiratory diseases
518.83	Chronic respiratory failure	Chronic respiratory diseases
518.84	Acute and chronic respiratory failure	Chronic respiratory diseases
414.8	Other specified forms of chronic ischemic heart disease	Chronic heart diseases
414.9	Unspecified chronic ischemic heart disease	Chronic heart diseases
428.22	Chronic systolic heart failure	Chronic heart diseases
428.23	Acute and chronic systolic heart failure	Chronic heart diseases
428.32	Chronic diastolic heart failure	Chronic heart diseases
428.33	Acute and chronic diastolic heart failure	Chronic heart diseases
428.42	Chronic combined systolic and diastolic heart failure	Chronic heart diseases
428.43	Acute and chronic combined systolic and diastolic heart failure	Chronic heart diseases
404.11	Benign hypertensive cardionephropathy with heart failure and chronic	Chronic kidney diseases
585.1	Chronic kidney disease, stage I	Chronic kidney diseases
585.2	Chronic kidney disease, stage II (mild)	Chronic kidney diseases
585.3	Chronic kidney disease, stage III (moderate)	Chronic kidney diseases
585.4	Chronic kidney disease, stage IV (severe)	Chronic kidney diseases
585.5	Stage V chronic kidney disease	Chronic kidney diseases
585.6	Chronic kidney disease, final stage	Chronic kidney diseases
585.9	Chronic kidney disease, unspecified	Chronic kidney diseases
250.00	Diabetes mellitus, type II or unspecified, undefined if decompensated,	Diabetes
250.01	Diabetes mellitus, type I (juvenile diabetes), undefined if	Diabetes
250.02	Diabetes mellitus, type II or unspecified, decompensated, without	Diabetes
250.03	Diabetes mellitus, type I (juvenile diabetes), decompensated	Diabetes
250.10	Diabetes with ketoacidosis, type II or unspecified, undefined if offset	Diabetes
250.11	Diabetes with ketoacidosis, type I (juvenile diabetes), not defined if	Diabetes
250.12	Diabetes with ketoacidosis, type II or unspecified, decompensated	Diabetes
250.13	Diabetes with ketoacidosis, type I (juvenile diabetes), decompensated	Diabetes
250.20	Diabetes with hyperosmolarity, type II or unspecified, undefined if	Diabetes
250.21	Diabetes with hyperosmolarity type I (juvenile diabetes), undefined if	Diabetes
250.22	Diabetes with hyperosmolarity, type II or unspecified, decompensated	Diabetes
250.30	Diabetes with other types of coma, type II or unspecified, undefined if	Diabetes
250.32	Diabetes with other types of coma, type II or unspecified,	Diabetes
250.40	Type II or unspecified diabetes, undefined if decompensated, with	Diabetes
250.41	Type I diabetes (juvenile diabetes), undefined if decompensated, with	Diabetes
250.42	Type II or unspecified diabetes, decompensated, with renal	Diabetes
250.43	Type I diabetes (juvenile diabetes), decompensated, with renal	Diabetes
250.50	Type II or unspecified diabetes, undefined if controlled, with ocular	Diabetes
250.51	Type I diabetes (juvenile diabetes), undefined when controlled, with	Diabetes
250.52	Type II or unspecified diabetes, decompensated, with eye	Diabetes
250.60	Type II or unspecified diabetes, undefined if decompensated, with	Diabetes
250.61	Type I diabetes (juvenile diabetes), undefined if decompensated, with	Diabetes
250.62	Type II or unspecified diabetes, decompensated, with neurological	Diabetes
250.63	Type I diabetes (juvenile diabetes), decompensated, with neurological	Diabetes
250.70	Type II or unspecified diabetes, undefined if decompensated, with	Diabetes

250.71	Type I diabetes (juvenile diabetes), undefined if decompensated, with	Diabetes
250.72	Type II or unspecified diabetes, decompensated, with peripheral	Diabetes
250.73	Type I diabetes (juvenile diabetes), decompensated, with peripheral	Diabetes
250.80	Type II or unspecified diabetes, undefined if decompensated, with	Diabetes
250.82	Type II or unspecified diabetes, decompensated, with other specified	Diabetes
250.90	Type II or unspecified diabetes, undefined if decompensated, with	Diabetes
250.91	Type I diabetes (juvenile diabetes), undefined if decompensated, with	Diabetes
250.92	Type II or unspecified diabetes, decompensated, with complications	Diabetes
253.5	Diabetes insipidus	Diabetes
357.2	Polyneuropathy in diabetes	Diabetes
366.41	Diabetic cataract	Diabetes
588.1	Nephrogenic diabetes insipidus	Diabetes
648.00	Diabetes mellitus, unspecified episode of cure	Diabetes
775.1	Neonatal diabetes mellitus	Diabetes
V18.0	Family history of diabetes mellitus	Diabetes
140.	Malignant tumors of the lips	Cancer
140.0	Malignant tumors of the upper lip, rosy border	Cancer
140.1	Malignant tumors of the lower lip, rosy border	Cancer
140.3	Malignant tumors of the upper lip, inner surface	Cancer
140.4	Malignant tumors of the lower lip, inner surface	Cancer
140.5	Malignant tumors of the unspecified lip, inner surface	Cancer
140.6	Malignant tumors of the commissure of the lips	Cancer
140.8	Malignant tumors of other sites of the lips	Cancer
140.9	Malignant lip tumors not specified, rosy border	Cancer
141.	Malignant tumors of the tongue	Cancer
141.0	Malignant tumors of the base of the tongue	Cancer
141.1	Malignant tumors of the dorsal surface of the tongue	Cancer
141.2	Malignant tumors of the tip and lateral margins of the tongue	Cancer
141.3	Malignant tumors of the ventral surface of the tongue	Cancer
141.4	Malignancies of the anterior two-thirds of the tongue, part unspecified	Cancer
141.5	Malignant tumors of the junctional zone	Cancer
141.6	Malignant tumors of the lingual tonsil	Cancer
141.8	Malignancies of other sites of the tongue	Cancer
141.9	Malignant tumors of the tongue, site not specified	Cancer
142.	Malignant tumors of the major salivary glands	Cancer
142.0	Malignant tumors of the parotid gland	Cancer
142.1	Malignant tumors of the submandibular gland	Cancer
142.2	Malignant tumors of the sublingual gland	Cancer
142.8	Malignant tumors of other major salivary glands	Cancer
142.9	Malignant tumors of the salivary gland, unspecified	Cancer
143.	Malignant tumors of the gums	Cancer
143.0	Malignant tumors of the upper gum	Cancer
143.1	Malignant tumors of the lower gum	Cancer
143.8	Malignant tumors of other sites of the gum	Cancer
143.9	Malignant tumors of the gum, unspecified	Cancer
144.	Malignant tumors of the floor of the mouth	Cancer
144.0	Malignant tumors of the front of the mouth	Cancer
144.1	Malignant tumors of the lateral part of the mouth	Cancer
144.8	Malignant tumors of other sites of the floor of the mouth	Cancer
144.9	Malignant tumors of the floor of the mouth, part not specified	Cancer
145.	Malignant tumors of other and unspecified parts of the mouth	Cancer
145.0	Malignant tumors of the mucous membrane of the cheeks	Cancer
145.1	Malignant tumors of the vestibule of the mouth	Cancer
145.2	Malignant tumors of the hard palate	Cancer
145.3	Malignant tumors of the soft palate	Cancer
145.4	Malignant tumors of the uvula	Cancer
145.5	Malignant tumors of the palate, unspecified	Cancer
145.6	Malignant tumors of the retromolar area	Cancer
145.8	Malignant tumors of other (specified) parts of the mouth	Cancer
145.9	Malignant tumors of the mouth, unspecified	Cancer

146.	Malignant tumors of the oropharynx	Cancer
146.0	Malignant tumors of the tonsil	Cancer
146.1	Malignant tumors of the tonsillar fossa	Cancer
146.2	Malignant tumors of the tonsillar pillars (anterior) (posterior)	Cancer
146.3	Malignant tumors of vallecula	Cancer
146.4	Malignant tumors of the anterior face of the epiglottis	Cancer
146.5	Malignant tumors of the junctional region	Cancer
146.6	Malignant tumors of the lateral wall of the oropharynx	Cancer
146.7	Malignant tumors of the posterior wall of the oropharynx	Cancer
146.8	Malignancies of other (specified) sites of the oropharynx	Cancer
146.9	Malignant tumors of the oropharynx, unspecified	Cancer
147.	Malignant tumors of the nasopharynx	Cancer
147.0	Malignant tumors of the upper wall of the nasopharynx	Cancer
147.1	Malignant tumors of the posterior wall of the nasopharynx	Cancer
147.2	Malignant tumors of the lateral wall of the nasopharynx	Cancer
147.3	Malignant tumors of the anterior wall of the nasopharynx	Cancer
147.8	Malignancies of other specified sites of the nasopharynx	Cancer
147.9	Malignant tumors of the nasopharynx, unspecified	Cancer
148.	Malignant tumors of the hypopharynx	Cancer
148.0	Malignant tumors of the retrocricoid region	Cancer
148.1	Malignant tumors of the piriformis sinus	Cancer
148.2	Malignant tumors of the hypopharyngeal face of the ariepigloptic fold	Cancer
148.3	Malignant tumors of the posterior hypopharyngeal wall	Cancer
148.8	Malignancies of other (specified) sites of the hypopharynx	Cancer
148.9	Malignant tumors of the hypopharynx, unspecified	Cancer
149.	Malignant tumors of other and ill-defined sites of the lips, oral cavity	Cancer
149.0	Malignant tumors of the pharynx, unspecified	Cancer
149.1	Malignant tumors of Waldeyer's ring	Cancer
149.8	Other malignant tumors of other and ill-defined sites of the lips, oral	Cancer
149.9	Malignant tumors of ill-defined sites of the lips, oral cavity and pharynx	Cancer
150.	Malignant tumors of the esophagus	Cancer
150.0	Malignant tumors of the cervical esophagus	Cancer
150.1	Malignant tumors of the thoracic esophagus	Cancer
150.2	Malignant tumors of the abdominal esophagus	Cancer
150.3	Malignant tumors of the upper third of the esophagus	Cancer
150.4	Malignant tumors of the middle third of the esophagus	Cancer
150.5	Malignant tumors of the lower third of the esophagus	Cancer
150.8	Malignant tumors of another specified part of the esophagus	Cancer
150.9	Malignant tumors of the esophagus, unspecified	Cancer
151.	Malignant tumors of the stomach	Cancer
151.0	Malignant tumors of the cardia	Cancer
151.1	Malignant pylorus tumors	Cancer
151.2	Malignant tumors of the pyloric antrum	Cancer
151.3	Malignant tumors of the bottom of the stomach	Cancer
151.4	Malignant tumors of the body of the stomach	Cancer
151.5	Malignant tumors of small curvature, unspecified	Cancer
151.6	Malignant tumors of the large curvature, unspecified	Cancer
151.8	Malignant tumors of other (specified) sites of the stomach	Cancer
151.9	Malignant tumors of the stomach, unspecified	Cancer
152.	Malignant tumors of the small intestine, including the duodenum	Cancer
152.0	Malignant tumors of the duodenum	Cancer
152.1	Malignant tumors of fasting	Cancer
152.2	Malignant tumors of the ileum	Cancer
152.3	Malignant tumors of Meckel's diverticulum	Cancer
152.8	Malignancies of other (specified) sites of the small intestine	Cancer
152.9	Malignant tumors of the small intestine, unspecified	Cancer
153.	Malignant tumors of the colon	Cancer
153.0	Malignant tumors of the hepatic flexure	Cancer
153.1	Malignant tumors of the transverse colon	Cancer
153.2	Malignant tumors of the descending colon	Cancer

153.3	Malignant tumors of the sigmoid	Cancer
153.4	Malignant tumors of the Czech	Cancer
153.5	Malignant tumors of the appendix	Cancer
153.6	Malignant tumors of the ascending colon	Cancer
153.7	Malignant tumors of splenic flexure	Cancer
153.8	Malignancies of other (specified) sites of the large intestine	Cancer
153.9	Malignant tumors of the colon, unspecified	Cancer
154.	Malignant tumors of the rectum, rectosigmoid junction and anus	Cancer
154.0	Malignant tumors of the rectosigmoid junction	Cancer
154.1	Malignant tumors of the rectum	Cancer
154.2	Malignant tumors of the canal	Cancer
154.3	Malignant tumors of the anus, unspecified	Cancer
154.8	Other malignant tumors of the rectum, rectosigmoid junction and anus	Cancer
155.	Malignant tumors of the liver and intrahepatic bile ducts	Cancer
155.0	Primary malignant tumors of the liver	Cancer
155.1	Malignant tumors of the intrahepatic bile ducts	Cancer
155.2	Malignant tumors of the liver, unspecified whether primary or	Cancer
156.	Malignant tumors of the gallbladder and extrahepatic bile ducts	Cancer
156.0	Malignant tumors of the gallbladder	Cancer
156.1	Malignant tumors of the extrahepatic bile ducts	Cancer
156.2	Malignant tumors of the ampulla of Vater	Cancer
156.8	Malignancies of other (specified) sites of the gallbladder and	Cancer
156.9	Malignant tumors of the biliary tract, unspecified	Cancer
157.	Malignant tumors of the pancreas	Cancer
157.0	Malignant tumors of the head of the pancreas	Cancer
157.1	Malignant tumors of the body of the pancreas	Cancer
157.2	Malignant tumors of the tail of the pancreas	Cancer
157.3	Malignant tumors of the pancreatic duct	Cancer
157.4	Malignant tumors of the islets of Langerhans	Cancer
157.8	Malignant tumors of other (specified) sites of the pancreas	Cancer
157.9	Malignant tumors of the pancreas, unspecified part	Cancer
158.	Malignant tumors of the retroperitoneum and peritoneum	Cancer
158.0	Malignant tumors of the retroperitoneum	Cancer
158.8	Malignant tumors of specified parts of the peritoneum	Cancer
158.9	Malignant tumors of the peritoneum, unspecified	Cancer
159.	Malignant tumors of other and ill-defined sites of the digestive system	Cancer
159.0	Malignant tumors of the intestinal tract, part not specified	Cancer
159.1	Malignant tumors of the spleen, not elsewhere classified	Cancer
159.8	Malignant tumors of other sites of the digestive system and	Cancer
159.9	Malignant tumors of poorly defined sites of the digestive system and	Cancer
160.	Malignant tumors of the nasal cavities, middle ear and paranasal	Cancer
160.0	Malignant tumors of the nasal cavities	Cancer
160.1	Malignant tumors of the auditory tube, middle ear and mastoid cells	Cancer
160.2	Malignant tumors of the maxillary sinus	Cancer
160.3	Malignant tumors of the ethmoid sinus	Cancer
160.4	Malignant tumors of the frontal sinus	Cancer
160.5	Malignant tumors of the sphenoid sinus	Cancer
160.8	Other malignancies of the nasal cavities, middle ear and paranasal	Cancer
160.9	Malignant tumors of the paranasal sinuses, unspecified	Cancer
161.	Malignant tumors of the larynx	Cancer
161.0	Malignant tumors of the glottis	Cancer
161.1	Malignant tumors of the supraglottic region	Cancer
161.2	Malignant tumors of the subglottic region	Cancer
161.3	Malignant tumors of laryngeal cartilage	Cancer
161.8	Malignancies of other specified sites of the larynx	Cancer
161.9	Malignant tumors of the larynx, unspecified	Cancer
162.	Malignant tumors of the trachea, bronchi and lungs	Cancer
162.0	Malignant tumors of the trachea	Cancer
162.2	Malignant tumors of the main bronchus	Cancer
162.3	Malignant tumors of the upper lobe, bronchus or lung	Cancer

162.4	Malignant tumors of the middle lobe, bronchus or lung	Cancer
162.5	Malignant tumors of the lower lobe, bronchus or lung	Cancer
162.8	Malignant tumors of other parts of the bronchi or lungs	Cancer
162.9	Malignant tumors of the bronchus or lung, unspecified	Cancer
163.	Malignant tumors of the pleura	Cancer
163.0	Malignant tumors of the parietal pleura	Cancer
163.1	Malignant tumors of the visceral pleura	Cancer
163.8	Malignancies of other specified sites of the pleura	Cancer
163.9	Malignant tumors of the pleura, unspecified	Cancer
164.	Malignant tumors of the thymus, heart and mediastinum	Cancer
164.0	Malignant tumors of the thymus	Cancer
164.1	Malignant tumors of the heart	Cancer
164.2	Malignant tumors of the anterior mediastinum	Cancer
164.3	Malignant tumors of the posterior mediastinum	Cancer
164.8	Other malignant tumors of the thymus, heart and mediastinum	Cancer
164.9	Malignant tumors of the mediastinum, unspecified	Cancer
165.	Malignant tumors of other and ill-defined sites of the respiratory system	Cancer
165.0	Malignant upper respiratory tract tumors, part not specified	Cancer
165.8	Other malignancies of other and ill-defined sites of the respiratory	Cancer
165.9	Malignant tumors of poorly defined sites of the respiratory system	Cancer
170.	Malignant tumors of bone and articular cartilage	Cancer
170.0	Malignant tumors of the bones of the skull and face, excluding the jaw	Cancer
170.1	Malignant tumors of the jaw	Cancer
170.2	Malignant tumors of the spine, excluding the sacrum and coccyx	Cancer
170.3	Malignant tumors of ribs, sternum and clavicle	Cancer
170.4	Malignant tumors of the scapula and long bones of the upper limb	Cancer
170.5	Malignant tumors of the short bones of the upper limb	Cancer
170.6	Malignant tumors of the pelvis, sacrum and coccyx	Cancer
170.7	Malignant tumors of the long bones of the lower limb	Cancer
170.8	Malignant tumors of the short bones of the lower limb	Cancer
170.9	Malignant tumors of bone and articular cartilage, site unspecified	Cancer
171.	Malignant tumors of connective tissue and other soft tissues	Cancer
171.0	Malignant tumors of the head, face and neck	Cancer
171.2	Malignant tumors of the upper limb including the shoulder	Cancer
171.3	Malignant tumors of the lower limb, including the hip	Cancer
171.4	Malignant tumors of the chest	Cancer
171.5	Malignant tumors of the abdomen	Cancer
171.6	Malignant tumors of the pelvis	Cancer
171.7	Malignant tumors of the trunk, unspecified	Cancer
171.8	Malignancies of other specified sites of connective tissue and other	Cancer
171.9	Malignant tumors of connective tissue and other soft tissues, site	Cancer
172.	Malignant melanoma of the skin	Cancer
172.0	Malignant melanoma of the lips	Cancer
172.1	Malignant melanoma of the eyelid, including the eyelid angle	Cancer
172.2	Malignant melanoma of ear and external ear canal	Cancer
172.3	Malignant melanoma of other and unspecified parts of the face	Cancer
172.4	Malignant melanoma of scalp and neck	Cancer
172.5	Malignant melanoma of the trunk, except the scrotum	Cancer
172.6	Malignant melanoma of the upper limb, including the shoulder	Cancer
172.7	Malignant melanoma of the lower limb, including hip	Cancer
172.8	Malignant melanoma of other specified sites of the skin	Cancer
172.9	Cutaneous melanoma, site unspecified	Cancer
173.	Other malignancies of the skin	Cancer
173.0	Other malignant tumors of the skin of the lips	Cancer
173.1	Other malignancies of the eyelid, including the eyelid angle	Cancer
173.2	Other malignant tumors of the skin of the ear and external ear canal	Cancer
173.3	Other malignant skin tumours of other and unspecified parts of the	Cancer
173.4	Other malignant tumors of the scalp and neck	Cancer
173.5	Other malignant tumors of the skin of the trunk, except the scrotum	Cancer
173.6	Other malignant tumors of the skin of the upper limb, including the	Cancer

173.7	Other malignancies of the skin of the lower limb, including the hip	Cancer
173.8	Other malignancies from other specified sites of the skin	Cancer
173.9	Other malignancies of the skin, site unspecified	Cancer
174.	Malignant tumors of the woman's breast	Cancer
174.0	Malignant tumors of the nipple and areola of the woman's breast	Cancer
174.1	Malignant tumors of the central part of the woman's breast	Cancer
174.2	Malignant tumors of the suprao-internal quadrant of the woman's	Cancer
174.3	Malignant tumors of the inferior-internal quadrant of the woman's	Cancer
174.4	Malignant tumors of the supra-external quadrant of the woman's breast	Cancer
174.5	Malignant tumors of the infero-external quadrant of the woman's breast	Cancer
174.6	Malignant tumors of axillary prolongation of the woman's breast	Cancer
174.8	Malignancies of the other specified sites of the woman's breast	Cancer
174.9	Malignant breast (female) tumors, unspecified	Cancer
175.	Malignant tumors of the human breast	Cancer
175.0	Malignant tumors of the nipple and areola of the human breast	Cancer
175.9	Malignancies of other and unspecified sites of the human breast	Cancer
176.	Kaposi's sarcoma	Cancer
176.0	Kaposi's sarcoma of the skin	Cancer
176.1	Kaposi's sarcoma of soft tissues	Cancer
176.2	Kaposi's sarcoma of the palate	Cancer
176.3	Kaposi's sarcoma of gastrointestinal sites	Cancer
176.4	Kaposi's sarcoma of the lung	Cancer
176.5	Kaposi's sarcoma of the lymph nodes	Cancer
176.8	Kaposi's sarcoma from other specified sites	Cancer
176.9	Kaposi's sarcoma not specified	Cancer
179.	Malignant tumors of the uterus, unspecified part	Cancer
180.	Malignant tumors of the cervix (uterine cervix)	Cancer
180.0	Malignant tumors of the endocervix	Cancer
180.1	Malignant tumors of the exocervix	Cancer
180.8	Malignant tumors of other specified sites of the cervix	Cancer
180.9	Malignant tumors of the uterine cervix, unspecified	Cancer
181.	Malignant tumors of the placenta	Cancer
182.	Malignant tumors of the body of the uterus	Cancer
182.0	Malignant tumors of the body of the uterus, except the isthmus	Cancer
182.1	Malignant tumors of the isthmus	Cancer
182.8	Malignant tumors of other specified sites of the body of the uterus	Cancer
183.	Malignant tumors of the ovary and other uterine appendages	Cancer
183.0	Malignant tumors of the ovary	Cancer
183.2	Malignant tumors of the fallopian tube	Cancer
183.3	Malignant tumors of the wide ligament	Cancer
183.4	Malignant tumors of the parametrium	Cancer
183.5	Malignant tumors of the round ligament	Cancer
183.8	Malignancies of other specified sites of uterine appendages	Cancer
183.9	Malignant tumors of uterine annexes, unspecified	Cancer
184.	Malignant tumors of other and unspecified female genital organs	Cancer
184.0	Malignant tumors of the vagina	Cancer
184.1	Malignant tumors of the labia majora	Cancer
184.2	Malignant tumors of the labia minora	Cancer
184.3	Malignant tumors of the clitoris	Cancer
184.4	Malignant tumors of the vulva, unspecified	Cancer
184.8	Malignancies of other specified locations of the female genital organs	Cancer
184.9	Malignancies of the female genital organs, site unspecified	Cancer
185.	Malignant tumors of the prostate	Cancer
186.	Malignant tumors of the testicle	Cancer
186.0	Malignant tumors of the testicle believed to be	Cancer
186.9	Malignant tumors of the testicle: other and unspecified	Cancer
187.	Malignant tumors of the penis and other male genital organs	Cancer
187.1	Malignant tumors of the foreskin	Cancer
187.2	Malignant tumors of the glans penis	Cancer
187.3	Malignant tumors of the body of the penis	Cancer

187.4	Malignant tumors of the penis, part not specified	Cancer
187.5	Malignant tumors of the epididymis	Cancer
187.6	Malignant tumors of the spermatic cord	Cancer
187.7	Malignant tumors of the scrotum	Cancer
187.8	Malignancies of other specified sites of the male genital organs	Cancer
187.9	Malignancies of the male genital organs, site unspecified	Cancer
188.	Malignant bladder tumors	Cancer
188.0	Malignant tumors of the bladder trine	Cancer
188.1	Malignant tumors of the bladder dome	Cancer
188.2	Malignant tumors of the lateral wall of the bladder	Cancer
188.3	Malignant tumors of the anterior wall of the bladder	Cancer
188.4	Malignant tumors of the posterior wall of the bladder	Cancer
188.5	Malignant tumors of the bladder neck	Cancer
188.6	Malignant tumors of the ureteral ostium	Cancer
188.7	Malignant tumors of the uracus	Cancer
188.8	Malignancies from other specified bladder sites	Cancer
188.9	Malignant tumors of unspecified part of the bladder	Cancer
189.	Malignant tumors of the kidney and other and unspecified urinary	Cancer
189.0	Malignant tumors of the kidney, except the pelvis	Cancer
189.1	Malignant tumors of the renal pelvis	Cancer
189.2	Malignant tumors of the ureter	Cancer
189.3	Malignant tumors of the urethra	Cancer
189.4	Malignant tumors of the paraurethral glands	Cancer
189.8	Malignancies of other specified urinary organ sites	Cancer
189.9	Malignant tumors of the urinary organs, site not specified	Cancer
190.	Malignant tumors of the eye	Cancer
190.0	Malignant tumors of the eyeball, except conjunctiva, cornea, retina and	Cancer
190.1	Malignant tumors of the orbit	Cancer
190.2	Malignant tumors of the lacrimal gland	Cancer
190.3	Malignant tumors of the conjunctiva	Cancer
190.4	Malignant tumors of the cornea	Cancer
190.5	Malignant tumors of the retina	Cancer
190.6	Malignant tumors of the choroid	Cancer
190.7	Malignant tumors of the tear ducts	Cancer
190.8	Malignancies from other specified eye sites	Cancer
190.9	Malignant tumors of the eye, part not specified	Cancer
191.	Malignant tumors of the brain	Cancer
191.0	Malignant tumors of the brain, except lobes and ventricles	Cancer
191.1	Malignant tumors of the frontal lobe	Cancer
191.2	Malignant tumors of the temporal lobe	Cancer
191.3	Malignant tumors of the parietal lobe	Cancer
191.4	Malignant tumors of the occipital lobe	Cancer
191.5	Malignant tumors of the ventricles	Cancer
191.6	Malignant tumors of the cerebellum, SAI	Cancer
191.7	Malignant tumors of the brainstem	Cancer
191.8	Malignant tumors of other parts of the brain	Cancer
191.9	Malignant brain tumors, unspecified	Cancer
192.	Malignant tumors of other and unspecified parts of the nervous system	Cancer
192.0	Malignant tumors of the cranial nerves	Cancer
192.1	Malignant tumors of the cerebral meninges	Cancer
192.2	Malignant tumors of the spinal cord	Cancer
192.3	Malignant tumors of the spinal meninges	Cancer
192.8	Malignancies of other specified sites of the nervous system	Cancer
192.9	Malignant tumors of the nervous system, part not specified	Cancer
193.	Malignant tumors of the thyroid gland	Cancer
194.	Malignant tumors of the other endocrine glands and related structures	Cancer
194.0	Malignant tumors of the adrenal glands	Cancer
194.1	Malignant tumors of the parathyroid glands	Cancer
194.3	Malignant tumors of the pituitary gland and craniopharyngeal duct	Cancer
194.4	Malignant tumors of the pineal gland	Cancer

194.5	Malignant tumors of the carotid glomus	Cancer
194.6	Malignant tumors of the aortic glomus and other paraganglia	Cancer
194.8	Other malignancies of the other endocrine glands and related	Cancer
194.9	Malignant tumors of the endocrine glands, site not specified	Cancer
195.	Malignant tumors of other and ill-defined sites	Cancer
195.0	Malignant tumors of the head, face and neck	Cancer
195.1	Malignant tumors of the chest	Cancer
195.2	Malignant tumors of the abdomen	Cancer
195.3	Malignant tumors of the pelvis	Cancer
195.4	Malignant tumors of the upper limb	Cancer
195.5	Malignant tumors of the lower limb	Cancer
195.8	Malignancies from other specified sites	Cancer
196.	Secondary and unspecified malignant tumors of lymph nodes	Cancer
196.0	Secondary and unspecified malignant tumors of the head, face and	Cancer
196.1	Secondary and unspecified malignant tumors of intrathoracic lymph	Cancer
196.2	Secondary and unspecified malignant tumors of intraabdominal lymph	Cancer
196.3	Secondary and unspecified malignant tumors of the lymph nodes of	Cancer
196.5	Secondary and unspecified malignant tumors of the lymph nodes of	Cancer
196.6	Secondary and unspecified malignant tumors of intrapelvic lymph	Cancer
196.8	Secondary and unspecified malignant tumors of lymph nodes, multiple	Cancer
196.9	Secondary and unspecified malignant tumors of lymph nodes, site	Cancer
197.	Secondary malignant tumors of the respiratory and digestive systems	Cancer
197.0	Secondary malignant tumors of the lung	Cancer
197.1	Secondary malignant tumors of the mediastinum	Cancer
197.2	Secondary malignant tumors of the pleura	Cancer
197.3	Secondary malignant tumors of other respiratory organs	Cancer
197.4	Secondary malignant tumors of the small intestine, including the	Cancer
197.5	Secondary malignant tumors of the colon and rectum	Cancer
197.6	Secondary malignant tumors of retroperitoneum and peritoneum	Cancer
197.7	Secondary malignancies of the liver, specified as metastatic	Cancer
197.8	Secondary malignant tumors of other digestive organs and spleen	Cancer
198.	Secondary malignancies from other specified sites	Cancer
198.0	Secondary malignant tumors of the kidney	Cancer
198.1	Secondary malignant tumors of other urinary organs	Cancer
198.2	Secondary malignant skin tumors	Cancer
198.3	Secondary malignant tumors of the brain and spinal cord	Cancer
198.4	Secondary malignancies of other parts of the nervous system	Cancer
198.5	Secondary malignant tumors of bone and bone marrow	Cancer
198.6	Secondary malignant tumors of the ovary	Cancer
198.7	Secondary malignant tumors of the adrenal gland	Cancer
198.8	Secondary malignancies from other specified sites	Cancer
198.81	Secondary malignant tumors of the breast	Cancer
198.82	Secondary malignant tumors of the genital organs	Cancer
198.89	Secondary malignancies from other specified sites	Cancer
199.	Malignancies without location indication	Cancer
199.0	Disseminated malignancies without location indication	Cancer
199.1	Other malignancies without indication of location	Cancer
200.	Lymphosarcoma and reticulosarcoma	Cancer
200.0	Reticulosarcoma	Cancer
200.00	Reticulosarcoma, site unspecified, solid organs or extranodal sites	Cancer
200.01	Reticulosarcoma, lymph nodes of the head and neck	Cancer
200.02	Reticulosarcoma, intrathoracic lymph nodes, mediastinal	Cancer
200.03	Reticulosarcoma, intraabdominal lymph nodes	Cancer
200.04	Reticulosarcoma, axillary lymph nodes and upper limb	Cancer
200.05	Reticulosarcoma, inguinal and lower limb lymph nodes	Cancer
200.06	Reticulosarcoma, pelvic lymph nodes	Cancer
200.07	Reticulossarcoma, spleen	Cancer
200.08	Reticulosarcoma, lymph nodes of multiple sites	Cancer
200.1	Lymphosarcoma	Cancer
200.10	Lymphosarcoma, site unspecified, solid organs or extranodal sites	Cancer

200.11	Lymphosarcoma, lymph nodes of the head and neck	Cancer
200.12	Lymphosarcoma, intrathoracic lymph nodes, mediastinal	Cancer
200.13	Lymphosarcoma, intraabdominal lymph nodes	Cancer
200.14	Lymphosarcoma, axillary lymph nodes and upper limb	Cancer
200.15	Lymphosarcoma, inguinal and lower limb lymph nodes	Cancer
200.16	Lymphosarcoma, pelvic lymph nodes	Cancer
200.17	Lymphosarcoma, spleen	Cancer
200.18	Lymphosarcoma, lymph nodes of multiple sites	Cancer
200.2	Lymphoma or Burkitt's tumor	Cancer
200.20	Lymphoma or Burkitt's tumor, site not specified, solid organs or	Cancer
200.21	Lymphoma or Burkitt's tumor, lymph nodes of the head and neck	Cancer
200.22	Lymphoma or Burkitt's tumor, intrathoracic lymph nodes, mediastinal	Cancer
200.23	Lymphoma or Burkitt's tumor, intraabdominal lymph nodes	Cancer
200.24	Lymphoma or Burkitt's tumor, axillary lymph nodes and upper limb	Cancer
200.25	Lymphoma or Burkitt's tumor, inguinal and lower limb lymph nodes	Cancer
200.26	Lymphoma or Burkitt's tumor, pelvic lymph nodes	Cancer
200.27	Lymphoma or Burkitt's tumor, spleen	Cancer
200.28	Lymphoma or Burkitt's tumor, lymph nodes of multiple sites	Cancer
200.8	Other variants	Cancer
200.80	Other variants, site not specified, solid organs or extranodal sites	Cancer
200.81	Other variants, lymph nodes of the head and neck	Cancer
200.82	Other variants, intrathoracic lymph nodes, mediastinal	Cancer
200.83	Other variants, intraabdominal lymph nodes	Cancer
200.84	Other variants, axillary lymph nodes and upper limb	Cancer
200.85	Other variants, inguinal and lower limb lymph nodes	Cancer
200.86	Other variants, pelvic lymph nodes	Cancer
200.87	Other variants, spleen	Cancer
200.88	Other variants, lymph nodes of multiple sites	Cancer
201.	Hodgkin's disease	Cancer
201.0	Hodgkin's paragranuloma	Cancer
201.00	Hodgkin's paragranuloma, site not specified, solid organs or extranodal	Cancer
201.01	Hodgkin's paragranuloma, lymph nodes of the head and neck	Cancer
201.02	Hodgkin's paragranuloma, intrathoracic, mediastinal lymph nodes	Cancer
201.03	Hodgkin's paragranuloma, intraabdominal lymph nodes	Cancer
201.04	Hodgkin's paragranuloma, axillary lymph nodes and upper limb	Cancer
201.05	Hodgkin's paragranuloma, inguinal and lower limb lymph nodes	Cancer
201.06	Hodgkin's paragranuloma, pelvic lymph nodes	Cancer
201.07	Hodgkin's paragranuloma, spleen	Cancer
201.08	Hodgkin's paragranuloma, lymph nodes of multiple sites	Cancer
201.1	Hodgkin's granuloma	Cancer
201.10	Hodgkin's granuloma, site not specified, solid organs or extranodal	Cancer
201.11	Hodgkin's granuloma, lymph nodes of the head and neck	Cancer
201.12	Hodgkin's granuloma, intrathoracic lymph nodes, mediastinal	Cancer
201.13	Hodgkin's granuloma, intraabdominal lymph nodes	Cancer
201.14	Hodgkin's granuloma, axillary lymph nodes and upper limb	Cancer
201.15	Hodgkin's granuloma, inguinal lymph nodes and lower limb	Cancer
201.16	Hodgkin's granuloma, pelvic lymph nodes	Cancer
201.17	Hodgkin's granuloma, spleen	Cancer
201.18	Hodgkin's granuloma, lymph nodes of multiple sites	Cancer
201.2	Hodgkin's sarcoma	Cancer
201.20	Hodgkin's sarcoma, site not specified, solid organs or extranodal sites	Cancer
201.21	Hodgkin's sarcoma, lymph nodes of the head and neck	Cancer
201.22	Hodgkin's sarcoma, intrathoracic lymph nodes, mediastinal	Cancer
201.23	Hodgkin's sarcoma, intraabdominal lymph nodes	Cancer
201.24	Hodgkin's sarcoma, axillary lymph nodes and upper limb	Cancer
201.25	Hodgkin's sarcoma, inguinal and lower limb lymph nodes	Cancer
201.26	Hodgkin's sarcoma, pelvic lymph nodes	Cancer
201.27	Hodgkin's sarcoma, spleen	Cancer
201.28	Hodgkin's sarcoma, lymph nodes of multiple sites	Cancer
201.4	Lymphocytic-histiocytic predominance	Cancer

201.40	Lymphocytic-histiocytic predominance, unspecified site, solid organs or	Cancer
201.41	Lymphocytic-histiocytic predominance, lymph nodes of the head and	Cancer
201.42	Lymphocytic-histiocytic predominance, intrathoracic, mediastinal lymph	Cancer
201.43	Lymphocytic-histiocytic predominance, intraabdominal lymph nodes	Cancer
201.44	Lymphocytic-histiocytic predominance, axillary lymph nodes and upper	Cancer
201.45	Lymphocytic-histiocytic predominance, inguinal and lower limb lymph	Cancer
201.46	Lymphocytic-histiocytic predominance, pelvic lymph nodes	Cancer
201.47	Lymphocytic-histiocytic predominance, spleen	Cancer
201.48	Lymphocytic-histiocytic predominance, lymph nodes of multiple sites	Cancer
201.5	Nodular sclerosis	Cancer
201.50	Nodular sclerosis, unspecified site, solid organs or extranodal sites	Cancer
201.51	Nodular sclerosis, lymph nodes of the head and neck	Cancer
201.52	Nodular sclerosis, intrathoracic lymph nodes, mediastinal	Cancer
201.53	Nodular sclerosis, intraabdominal lymph nodes	Cancer
201.54	Nodular sclerosis, axillary lymph nodes and upper limb	Cancer
201.55	Nodular sclerosis, inguinal and lower limb lymph nodes	Cancer
201.56	Nodular sclerosis, pelvic lymph nodes	Cancer
201.57	Nodular sclerosis, spleen	Cancer
201.58	Nodular sclerosis, lymph nodes of multiple sites	Cancer
201.6	Mixed cellularity	Cancer
201.60	Mixed cellularity, site not specified, solid organs or extranodal sites	Cancer
201.61	Mixed cellularity, lymph nodes of the head and neck	Cancer
201.62	Mixed cellularity, intrathoracic lymph nodes, mediastinal	Cancer
201.63	Mixed cellularity, intraabdominal lymph nodes	Cancer
201.64	Mixed cellularity, axillary and upper limb lymph nodes	Cancer
201.65	Mixed cellularity, inguinal and lower limb lymph nodes	Cancer
201.66	Mixed cellularity, pelvic lymph nodes	Cancer
201.67	Mixed cellularity, spleen	Cancer
201.68	Mixed cellularity, lymph nodes of multiple sites	Cancer
201.7	Lymphocyte depletion	Cancer
201.70	Lymphocyte depletion, site unspecified, solid organs or extranodal	Cancer
201.71	Lymphocyte depletion, lymph nodes of the head and neck	Cancer
201.72	Lymphocyte depletion, intrathoracic lymph nodes, mediastinal	Cancer
201.73	Lymphocyte depletion, intraabdominal lymph nodes	Cancer
201.74	Lymphocyte depletion, axillary lymph nodes and upper limb	Cancer
201.75	Lymphocyte depletion, inguinal lymph nodes and lower limb	Cancer
201.76	Lymphocyte depletion, pelvic lymph nodes	Cancer
201.77	Lymphocyte depletion, spleen	Cancer
201.78	Lymphocyte depletion, lymph nodes of multiple sites	Cancer
201.9	Hodgkin's disease, unspecified	Cancer
201.90	Hodgkin's disease, unspecified, site unspecified, solid organs or	Cancer
201.91	Hodgkin's disease, unspecified, lymph nodes of the head and neck	Cancer
201.92	Hodgkin's disease, unspecified, intrathoracic lymph nodes, mediastinal	Cancer
201.93	Hodgkin's disease, unspecified, intraabdominal lymph nodes	Cancer
201.94	Hodgkin's disease, unspecified, axillary and upper limb lymph nodes	Cancer
201.95	Hodgkin's disease, unspecified, inguinal and lower limb lymph nodes	Cancer
201.96	Hodgkin's disease, unspecified, pelvic lymph nodes	Cancer
201.97	Hodgkin's disease, unspecified, spleen	Cancer
201.98	Hodgkin's disease, unspecified, lymph nodes of multiple sites	Cancer
202.	Other malignancies of lymphatic and histiocytic tissue	Cancer
202.0	Nodular lymphoma	Cancer
202.00	Nodular lymphoma, unspecified site, solid organs or extranodal sites	Cancer
202.01	Nodular lymphoma, lymph nodes of the head and neck	Cancer
202.02	Nodular lymphoma, intrathoracic lymph nodes, mediastinal	Cancer
202.03	Nodular lymphoma, intraabdominal lymph nodes	Cancer
202.04	Nodular lymphoma, axillary lymph nodes and upper limb	Cancer
202.05	Nodular lymphoma, inguinal and lower limb lymph nodes	Cancer
202.06	Nodular lymphoma, pelvic lymph nodes	Cancer
202.07	Nodular lymphoma, spleen	Cancer
202.08	Nodular lymphoma, lymph nodes of multiple sites	Cancer

202.1	Mycosis fungoides	Cancer
202.10	Mycosis fungoides, site not specified, solid organs or extranodal sites	Cancer
202.11	Mycosis fungoides, lymph nodes of the head and neck	Cancer
202.12	Mycosis fungoides, intrathoracic, mediastinal lymph nodes	Cancer
202.13	Mycosis fungoides, intraabdominal lymph nodes	Cancer
202.14	Mycosis fungoides, axillary lymph nodes and upper limb	Cancer
202.15	Mycosis fungoides, inguinal lymph nodes and lower limb	Cancer
202.16	Mycosis fungoides, pelvic lymph nodes	Cancer
202.17	Mycosis fungoides, spleen	Cancer
202.18	Mycosis fungoides, lymph nodes of multiple sites	Cancer
202.2	Se¤zary disease	Cancer
202.20	Se¤zary disease, site not specified, solid organs or extranodal sites	Cancer
202.21	Se¤zary's disease, lymph nodes of the head and neck	Cancer
202.22	Se¤zary disease, intrathoracic lymph nodes, mediastinal	Cancer
202.23	Se¤zary's disease, intraabdominal lymph nodes	Cancer
202.24	Se¤zary's disease, axillary lymph nodes and upper limb	Cancer
202.25	Se¤zary's disease, inguinal and lower limb lymph nodes	Cancer
202.26	Se¤zary's disease, pelvic lymph nodes	Cancer
202.27	Se¤zary disease, spleen	Cancer
202.28	Se¤zary disease, lymph nodes of multiple sites	Cancer
202.3	Malignant histiocytosis	Cancer
202.30	Malignant histiocytosis, unspecified site, solid organs or extranodal	Cancer
202.31	Malignant histiocytosis, lymph nodes of the head and neck	Cancer
202.32	Malignant histiocytosis, intrathoracic, mediastinal lymph nodes	Cancer
202.33	Malignant histiocytosis, intraabdominal lymph nodes	Cancer
202.34	Malignant histiocytosis, axillary and upper limb lymph nodes	Cancer
202.35	Malignant histiocytosis, inguinal and lower limb lymph nodes	Cancer
202.36	Malignant histiocytosis, pelvic lymph nodes	Cancer
202.37	Malignant histiocytosis, spleen	Cancer
202.38	Malignant histiocytosis, lymph nodes of multiple sites	Cancer
202.4	Leukemic reticuloendotheliosis	Cancer
202.40	Leukemic reticuloendotheliosis, site unspecified, solid organs or	Cancer
202.41	Leukemic reticuloendotheliosis, lymph nodes of the head and neck	Cancer
202.42	Leukemic reticuloendotheliosis, intrathoracic lymph nodes, mediastinal	Cancer
202.43	Leukemic reticuloendotheliosis, intraabdominal lymph nodes	Cancer
202.44	Leukemic reticuloendotheliosis, axillary and upper limb lymph nodes	Cancer
202.45	Leukemic reticuloendotheliosis, inguinal and lower limb lymph nodes	Cancer
202.46	Leukemic reticuloendotheliosis, pelvic lymph nodes	Cancer
202.47	Leukemic reticuloendotheliosis, spleen	Cancer
202.48	Leukemic reticuloendotheliosis, lymph nodes of multiple sites	Cancer
202.5	Letterer Siwe disease	Cancer
202.50	Siwe Letterer disease, site not specified, solid organs or extranodal	Cancer
202.51	Letterer Siwe disease, lymph nodes of the head and neck	Cancer
202.52	Letterer Siwe disease, intrathoracic, mediastinal lymph nodes	Cancer
202.53	Letterer Siwe disease, intraabdominal lymph nodes	Cancer
202.54	Letterer Siwe's disease, axillary lymph nodes and upper limb	Cancer
202.55	Letterer Siwe disease, inguinal and lower limb lymph nodes	Cancer
202.56	Letterer Siwe disease, pelvic lymph nodes	Cancer
202.57	Letterer Siwe disease, spleen	Cancer
202.58	Letterer Siwe disease, lymph nodes of multiple sites	Cancer
202.6	Malignant tumors of mast cells	Cancer
202.60	Malignant mast cell tumors, site unspecified, solid organs or extranodal	Cancer
202.61	Malignant tumors of mast cells, lymph nodes of the head and neck	Cancer
202.62	Malignant tumors of mast cells, intrathoracic, mediastinal lymph nodes	Cancer
202.63	Malignant tumors of mast cells, intraabdominal lymph nodes	Cancer
202.64	Malignant tumors of mast cells, axillary lymph nodes and upper limb	Cancer
202.65	Malignant tumors of mast cells, inguinal lymph nodes and lower limb	Cancer
202.66	Malignant tumors of mast cells, pelvic lymph nodes	Cancer
202.67	Malignant tumors of mast cells, spleen	Cancer
202.68	Malignant tumors of mast cells, lymph nodes of multiple sites	Cancer

202.8	Other lymphomas	Cancer
202.80	Other lymphomas, site not specified, solid organs or extranodal sites	Cancer
202.81	Other lymphomas, lymph nodes of the head and neck	Cancer
202.82	Other lymphomas, intrathoracic lymph nodes, mediastinal	Cancer
202.83	Other lymphomas, intraabdominal lymph nodes	Cancer
202.84	Other lymphomas, axillary lymph nodes and upper limb	Cancer
202.85	Other lymphomas, inguinal lymph nodes and lower limb	Cancer
202.86	Other lymphomas, pelvic lymph nodes	Cancer
202.87	Other lymphomas, spleen	Cancer
202.88	Other lymphomas, lymph nodes of multiple sites	Cancer
202.9	Other unspecified malignancies of lymphoid and histiocytic tissue	Cancer
202.90	Other unspecified malignancies of lymphoid and histiocytic tissue,	Cancer
202.91	Other unspecified malignancies of lymphoid and histiocytic tissue,	Cancer
202.92	Other unspecified malignancies of lymphoid and histiocytic tissue,	Cancer
202.93	Other unspecified malignancies of lymphoid and histiocyte tissue,	Cancer
202.94	Other unspecified malignancies of lymphoid and histiocytic tissue,	Cancer
202.95	Other unspecified malignancies of lymphoid and histiocytic tissue,	Cancer
202.96	Other unspecified malignancies of lymphoid and histiocytic tissue,	Cancer
202.97	Other unspecified malignancies of lymphoid and histiocyte tissue,	Cancer
202.98	Other unspecified malignancies of lymphoid and histiocyte tissue,	Cancer
203.	Multiple myeloma and immunoproliferative malignancies	Cancer
203.0	Myeloma	Cancer
203.00	Multiple myeloma, no mention of remission	Cancer
203.01	Multiple myeloma, in remission	Cancer
203.1	Plasma cell leukemia	Cancer
203.10	Plasma cell leukemia, without mention of remission	Cancer
203.11	Plasma cell leukemia, in remission	Cancer
203.8	Other immunoproliferative malignancies	Cancer
203.80	Other immunoproliferative neoplasms, without mention of remission	Cancer
203.81	Other immunoproliferative neoplasms, in remission	Cancer
204.	Lymphoid leukemia	Cancer
204.0	Lymphoid leukemia, acute	Cancer
204.00	Lymphoid leukemia, acute, without mention of remission	Cancer
204.01	Lymphoid leukemia, acute, in remission	Cancer
204.1	Lymphoid leukemia, chronic	Cancer
204.10	Lymphoid leukemia, chronic, without mention of remission	Cancer
204.11	Lymphoid leukemia, chronic, in remission	Cancer
204.2	Lymphoid leukemia, subacute	Cancer
204.20	Lymphoid leukemia, subacute, without mention of remission	Cancer
204.21	Lymphoid leukemia, subacute, in remission	Cancer
204.8	Other lymphatic leukemias	Cancer
204.80	Other lymphatic leukemias, without mention of remission	Cancer
204.81	Other lymphatic leukemias, in remission	Cancer
204.9	Lymphoid leukemia, unspecified	Cancer
204.90	Lymphoid leukemia, unspecified, without mention of remission	Cancer
204.91	Lymphoid leukemia, unspecified, in remission	Cancer
205.	Myeloid leukemia	Cancer
205.0	Myeloid leukemia, acute	Cancer
205.00	Myeloid leukemia, acute, without mention of remission	Cancer
205.01	Myeloid leukemia, acute, in remission	Cancer
205.1	Myeloid leukemia, chronic	Cancer
205.10	Myeloid leukemia, chronic, without mention of remission	Cancer
205.11	Myeloid leukemia, chronic, in remission	Cancer
205.2	Myeloid leukemia, subacute	Cancer
205.20	Myeloid leukemia, subacute, without mention of remission	Cancer
205.21	Myeloid leukemia, subacute, in remission	Cancer
205.3	Myeloid sarcoma	Cancer
205.30	Myeloid sarcoma, without mention of remission	Cancer
205.31	Myeloid sarcoma, in remission	Cancer
205.8	Other myeloid leukemias	Cancer

205.80	Other myeloid leukemias, without mention of remission	Cancer
205.81	Other myeloid leukemias, in remission	Cancer
205.9	Myeloid leukemia, unspecified	Cancer
205.90	Myeloid leukemia, unspecified, without mention of remission	Cancer
205.91	Myeloid leukemia, unspecified, in remission	Cancer
206.	Monocytic leukemia	Cancer
206.0	Monocytic leukemia, acute	Cancer
206.00	Monocytic leukemia, acute, without mention of remission	Cancer
206.01	Monocytic leukemia, acute, in remission	Cancer
206.1	Monocytic leukemia, chronic	Cancer
206.10	Monocytic leukemia, chronic, without mention of remission	Cancer
206.11	Monocytic leukemia, chronic, in remission	Cancer
206.2	Monocytic leukemia, subacute	Cancer
206.20	Monocytic leukemia, subacute, without mention of remission	Cancer
206.21	Monocytic leukemia, subacute, in remission	Cancer
206.8	Other monocytic leukemias	Cancer
206.80	Other monocytic leukemias, without mention of remission	Cancer
206.81	Other monocytic leukemias, in remission	Cancer
206.9	Monocytic leukemia, unspecified	Cancer
206.90	Monocytic leukemia, unspecified, without mention of remission	Cancer
206.91	Monocytic leukemia, unspecified, in remission	Cancer
207.	Other specific leukemias	Cancer
207.0	Acute erythremia and erythroleukemia	Cancer
207.00	Acute erythremia and erythroleukemia, without mention of remission	Cancer
207.01	Acute erythremia and erythroleukemia, in remission	Cancer
207.1	Chronic erythremia	Cancer
207.10	Chronic erythremia, without mention of remission	Cancer
207.11	Chronic erythremia, in remission	Cancer
207.2	Megakaryocytic leukemia	Cancer
207.20	Megakaryocytic leukemia, without mention of remission	Cancer
207.21	Megakaryocytic leukemia, in remission	Cancer
207.8	Other specific leukemias	Cancer
207.80	Other specific leukemias, without mention of remission	Cancer
207.81	Other specific leukemias, in remission	Cancer
208.	Unspecified cell type leukemia	Cancer
208.0	Unspecified, acute cell type leukemia	Cancer
208.00	Unspecified cell type leukemia, acute, with no mention of remission	Cancer
208.01	Unspecified cell type leukemia, acute, in remission	Cancer
208.1	Unspecified, chronic cell type leukemia	Cancer
208.10	Unspecified, chronic cell type leukemia with no mention of remission	Cancer
208.11	Unspecified cell type leukemia, chronic, in remission	Cancer
208.2	Unspecified, subacute cell leukemia	Cancer
208.20	Unspecified, subacute cell type leukemia with no mention of remission	Cancer
208.21	Unspecified cell type leukemia, subacute, in remission	Cancer
208.8	Other acute cell type leukaemias not specified	Cancer
208.80	Other acute cell type leukaemias not specified, without mention of	Cancer
208.81	Other acute leukemias of unspecified cell type, acute, in remission	Cancer
208.9	Unspecified leukemia	Cancer
208.90	Leukemia not specified, without mention of remission	Cancer
208.91	Unspecified leukemia, in remission	Cancer
230.	In situ carcinomas of the digestive organs	Cancer
230.0	In situ carcinomas of the lips, oral cavity and pharynx	Cancer
230.1	In situ carcinomas of the esophagus	Cancer
230.2	In situ carcinomas of the stomach	Cancer
230.3	In situ carcinomas of the colon	Cancer
230.4	Carcinomas in situ of the rectum	Cancer
230.5	Carcinomas in situ of the canal	Cancer
230.6	In situ carcinomas of the anus, unspecified	Cancer
230.7	In situ carcinomas of other and unspecified sites of the intestine	Cancer
230.8	In situ carcinomas of liver and biliary tract	Cancer

230.9	In situ carcinomas of other and unspecified digestive organs	Cancer
231.	In situ carcinomas of the respiratory system	Cancer
231.0	In situ carcinomas of the larynx	Cancer
231.1	In situ carcinomas of the trachea	Cancer
231.2	In situ carcinomas of bronchi and lungs	Cancer
231.8	In situ carcinomas of other specified sites of the respiratory system	Cancer
231.9	In situ carcinomas of the respiratory system, site not specified	Cancer
232.	Carcinomas in situ of the skin	Cancer
232.0	Carcinomas in situ of the skin of the lips	Cancer
232.1	Carcinomas in situ of the eyelid, including the eyelid angle	Cancer
232.2	In situ carcinomas of ear and external ear canal	Cancer
232.3	In situ carcinomas of the skin of other and unspecified parts of the face	Cancer
232.4	In situ carcinomas of scalp and skin of the neck	Cancer
232.5	Carcinomas in situ of skin of the trunk, except the scrotum	Cancer
232.6	Carcinomas in situ of the skin of the upper limb, including the shoulder	Cancer
232.7	Carcinomas in situ of the skin of the lower limb, including the hip	Cancer
232.8	In situ carcinomas of other specified skin sites	Cancer
232.9	In situ skin carcinomas, site unspecified	Cancer
233.	In situ carcinomas of the breast and urogenital apparatus	Cancer
233.0	In situ breast carcinomas	Cancer
233.1	In situ carcinomas of the uterine cervix	Cancer
233.2	In situ carcinomas of other and unspecified parts of the uterus	Cancer
233.3	In situ carcinomas of other and unspecified female genital organs	Cancer
233.4	In situ carcinomas of the prostate	Cancer
233.5	Carcinomas in situ of the penis	Cancer
233.6	In situ carcinomas of other and unspecified male genital organs	Cancer
233.7	Carcinomas in situ of the bladder	Cancer
233.9	In situ carcinomas of other and unspecified urinary organs	Cancer
234.	In situ carcinomas of other and unspecified sites	Cancer
234.0	Carcinomas in situ of the eye	Cancer
234.8	In situ carcinomas of other specified sites	Cancer
234.9	Carcinomas in situ, site not specified	Cancer
235.	Tumors of uncertain behavior of the digestive and respiratory systems	Cancer
235.0	Tumors of uncertain behavior of the major salivary glands	Cancer
235.1	Tumors of uncertain behavior of the lips, oral cavity and pharynx	Cancer
235.2	Tumors of uncertain behavior of the stomach, intestines and rectum	Cancer
235.3	Tumors of uncertain behavior of liver and biliary tract	Cancer
235.4	Tumors of uncertain behavior of retroperitoneum and peritoneum	Cancer
235.5	Tumors of uncertain behavior of other and unspecified digestive	Cancer
235.6	Tumors of uncertain behavior of the larynx	Cancer
235.7	Tumors of uncertain behavior of trachea, bronchi and lungs	Cancer
235.8	Tumors of uncertain behavior of pleura, thymus and mediastinum	Cancer
235.9	Tumors of uncertain behavior of other and unspecified respiratory	Cancer
236.	Tumors of uncertain behavior of the urogenital organs	Cancer
236.0	Tumors of uncertain behavior of the uterus	Cancer
236.1	Tumors of uncertain placental behavior	Cancer
236.2	Tumors of uncertain ovarian behavior	Cancer
236.3	Tumors of uncertain behavior of other and unspecified female genital	Cancer
236.4	Tumors of uncertain testicular behavior	Cancer
236.5	Tumors of uncertain behavior of the prostate	Cancer
236.6	Tumors of uncertain behavior of other and unspecified male genital	Cancer
236.7	Tumors of uncertain bladder behavior	Cancer
236.9	Tumors of uncertain behavior of other and unspecified urinary organs	Cancer
236.90	Tumors of uncertain behavior of the urinary organs, unspecified	Cancer
236.91	Tumors of uncertain behavior of kidney and ureter	Cancer
236.99	Other tumors of uncertain behavior of the urogenital organs	Cancer
237.	Tumors of uncertain behavior of the endocrine glands and nervous	Cancer
237.0	Tumors of uncertain pituitary and craniopharyngeal duct behavior	Cancer
237.1	Tumors of uncertain behavior of the pineal gland	Cancer
237.2	Tumors of uncertain behavior of the adrenal glands	Cancer

237.3	Tumors of uncertain behavior of paraganglia	Cancer
237.4	Tumors of uncertain behavior of other and unspecified endocrine	Cancer
237.5	Tumors of uncertain behavior of the brain and spinal cord	Cancer
237.6	Tumors of uncertain behavior of the meninges	Cancer
237.7	Neurofibromatosis	Cancer
237.70	Neurofibromatosis, unspecified	Cancer
237.71	Neurofibromatosis type I [von Recklinghausen disease]	Cancer
237.72	Neurofibromatosis type II [acoustic neurofibromatosis]	Cancer
237.9	Tumors of uncertain behavior of other and unspecified parts of the	Cancer
238.	Tumors of uncertain behavior of other and unspecified tissues and	Cancer
238.0	Tumors of uncertain behavior of bone and articular cartilage	Cancer
238.1	Tumors of uncertain behavior of connective tissue and other soft	Cancer
238.2	Tumors of uncertain skin behavior	Cancer
238.3	Tumors of uncertain breast behavior	Cancer
238.4	Tumors of uncertain behavior of polycythemia vera	Cancer
238.5	Tumors of uncertain behavior of histiocytes and mast cells	Cancer
238.6	Tumors of uncertain plasma cell behavior	Cancer
238.7	Tumors of uncertain behavior of other lymphatic and hematopoietic	Cancer
238.71	Essential thrombocythemia	Cancer
238.72	Myelodysplastic syndrome with low-grade lesions	Cancer
238.73	Myelodysplastic syndrome with high-grade lesions	Cancer
238.74	Myelodysplastic syndrome with deletion	Cancer
238.75	Myelodysplastic syndrome, unspecified	Cancer
238.76	Myelofibrosis with myeloid metaplasia	Cancer
238.79	Other lymphatic and hematopoietic tissues	Cancer
238.8	Tumors of uncertain behavior from other specified sites	Cancer
238.9	Tumors of uncertain behavior, site not specified	Cancer
239.	Tumors of unspecified nature	Cancer
239.0	Tumors of unspecified nature of the digestive system	Cancer
239.1	Tumors of an unspecified nature of the respiratory system	Cancer
239.2	Tumors of unspecified nature of bone, soft tissue and skin	Cancer
239.3	Tumors of unspecified nature of the breast	Cancer
239.4	Tumors of unspecified nature of the bladder	Cancer
239.5	Tumors of an unspecified nature of other urogenital organs	Cancer
239.6	Tumors of unspecified nature of the brain	Cancer
239.7	Tumors of unspecified nature of the endocrine glands and other parts	Cancer
239.8	Tumors of an unspecified nature from other specified sites	Cancer
239.9	Tumors of unspecified nature, site not specified	Cancer
